# Network Analysis of the Systemic Response to *Fasciola hepatica* Infection in Sheep Reveals Changes in Fibrosis, Apoptosis, Toll-Like Receptors 3/4, and B Cell Function

**DOI:** 10.3389/fimmu.2017.00485

**Published:** 2017-04-25

**Authors:** Yan Fu, John A. Browne, Kate Killick, Grace Mulcahy

**Affiliations:** ^1^UCD School of Veterinary Medicine, University College Dublin, Dublin, Ireland; ^2^UCD School of Agriculture and Food Science, University College Dublin, Dublin, Ireland; ^3^UCD Conway Institute of Biomolecular and Biomedical Research, University College Dublin, Dublin, Ireland

**Keywords:** *Fasciola hepatica*, peripheral blood mononuclear cells, innate immunity, toll-like receptor, apoptosis, B-cell receptor

## Abstract

The Trematode *Fasciola hepatica* is an important cause of disease in livestock and in man. Modulation of immunity is a critical strategy used by this parasite to facilitate its long-term survival in the host. Understanding the underlying mechanisms at a system level is important for the development of novel control strategies, such as vaccination, as well as for increasing general understanding of helminth-mediated immunoregulation and its consequences. Our previous RNA sequencing work identified a large number of differentially expressed genes (DEG) from ovine peripheral blood mononuclear cells (PBMCs) at acute and chronic stages of *F. hepatica* infection, and yielded important information on host–parasite interaction, with particular reference to the immune response. To extend our understanding of the immunoregulatory effects of this parasite, we employed InnateDB to further analyze the DEG dataset and identified 2,458 and 224 molecular interactions in the context of innate immunity from the acute and chronic stages of infection, respectively. Notably, 458 interactions at the acute stage of infection were manually curated from studies involving PBMC-related cell-types, which guaranteed confident hypothesis generation. NetworkAnalyst was subsequently used to construct and visualize molecular networks. Two complementary strategies (function-first and connection-first) were conducted to interpret the networks. The function-first approach highlighted subnetworks implicated in regulation of Toll-like receptor 3/4 signaling in both acute and chronic infections. The connection-first approach highlighted regulation of intrinsic apoptosis and B-cell receptor-signaling during acute and chronic infections, respectively. To the best of our knowledge, this study is the first system level analysis of the regulation of host innate immunity during *F. hepatica* infection. It provides insights into the profound changes induced by *F. hepatica* infection that not only favors parasite survival into chronic infection but also impedes the host’s immune response to other pathogens, and render vaccination against fasciolosis a difficult challenge. The information provided will be useful in the design of specific vaccine protocols to overcome parasite-mediated immunoregulation and in furthering general understanding of the interplay between helminth infection and host immune systems.

## Introduction

*Fasciola hepatica* is not only an important helminth parasite of economically important animals, such as cattle and sheep (1), but also a major food-borne zoonosis worldwide (2–4). As chemotherapy is the major control mechanism available currently, with the most frequently used drug being triclabendazole, intensive use of this compound has directly resulted in development of drug resistance in the endemic areas across Europe, Australia, and some parts of South America (5–8). In addition, drug-based control also raises problems of chemical residues in food and in the environment, which has generated increasing consumer concern. Therefore, the development of novel control strategies, and particularly vaccination, is urgently needed. A better understanding of host–parasite interactions is expected to further refine development of vaccines and may lead to novel therapeutic approaches against *F. hepatica* infection.

As the first, and perhaps, the most critical line of defense against invading pathogens, the innate immune response plays a crucial role in the initiation of and interplay with the adaptive immune response (9). *F. hepatica* interacts with various host innate immune cells [e.g., dendritic cells (DC), macrophages, and mast cells] as soon as infection commences with excystation of the juvenile flukes which cross the gut wall within hours (10). Innate effector mechanisms, such as alternative activation of macrophages, play an important role in early host responses against *F. hepatica* infection as well as in regulating and shaping the Th2-biased adaptive immune response (11). Previous studies have also suggested that *F. hepatica* infection can modify the maturation and function of host DC through a number of different mechanisms, which could further mediate the stimulation of naïve T cells (12–14).

We have previously reported the transcriptomic changes of peripheral blood mononuclear cells (PBMCs) from eight lambs in response to *F. hepatica* infection using RNA sequencing (RNA-seq) (NCBI GEO accession number: GSE71431) (15). Two datasets, including 5,409 and 2,364 differentially expressed genes (DEG), respectively, were generated from eight biological replicates by comparisons between preinfection and week one postinfection (wpi), and between 1 and 14 wpi, representing transcript expression changes induced by acute and chronic infections, respectively. Further examination using ingenuity pathway analysis (IPA) revealed important insights into host–parasite interactions, such as regulation of fibrosis, nitric oxide production, apoptosis, and toll-like receptor (TLR) regulation.

InnateDB (http://www.innatedb.com/) is an integrated analysis tool that has been specifically designed to facilitate analysis of innate immunity gene interactions and networks (16). Network construction is useful for visualization and analysis of interactomes, by creating unbiased mechanistic hypotheses based on both node connectivity and expression changes observed in datasets (17, 18). Here, we extend our previous work by further analyzing RNA-seq data using InnateDB and NetworkAnalyst, which provided system-level understanding of the ovine innate immune response to *F. hepatica* infection. We generated 2,458 and 224 molecular interactions in the context of innate immunity from acute and chronic stages of infection, respectively. Notably, there were 458 interactions at the acute stage of infection generated from studies involving PBMC-related cell types.

## Materials and Methods

Molecular interactome analysis was carried out on the 5,409 and 2,364 DEG (FDR < 5%) detected in ovine PBMC during the acute and chronic stages of *F. hepatica* infection, respectively (15), using InnateDB (16). For mapping with the InnateDB interaction database, the option “Return InnateDB-curated interactions only” was chosen. Since the sheep is not currently a supported species in InnateDB, human orthologs for the relevant sheep genes were used. The human ortholog information was downloaded from biomaRt and merged with the significant genes. In cases where a single sheep gene was annotated to multiple human genes, just one human ortholog was retained for the purposes of pathway analysis.

DEG involved in immune/fibrosis-associated molecular interactions were manually picked out according to the InnateDB-recorded interaction evidence that refers to the experimental procedures and conditions (16). The selected DEG were subsequently used for network construction using NetworkAnalyst (19), which was also used for functional analysis on these networks. To reduce network complexity while keeping the most interesting functions and connections, we employed two heuristic approaches, function-first and connection-first (19), to interpret our data. In the first approach, we enriched the biological functions within the network followed by extracting subnetworks containing nodes that were involved in functions of interest, thereby guaranteeing that the resulting subnetworks are interpretable in the context of corresponding biological function. Subsequently, we performed module exploration analysis on the subnetwork of interest. The nodes within each module are likely to work together to perform a biological function (18). In the latter approach, we first used the module generation function built-in NetworkAnalyst to deconstruct the current network into smaller densely connected modules, which are considered as tight clusters of molecules with more internal connections than expected randomly in the network as a whole (18). Subsequently, each module was subject to functional enrichment analysis. This approach was used to guarantee high connectivity of the resulting modules. For both approaches, functional enrichment was performed by using the “Reactome” database. Reactome is an open-source, curated, and peer-reviewed pathway database of human biological processes (20), which has been integrated into the “Function Explorer” section of NetworkAnalyst.

The Wilcoxon rank-sum test was performed to calculate the significance of each module by testing the difference between the number of edges (edges represent interactions between two connecting nodes) within a module and the edges connecting the nodes of a module with the rest of the network (18). A hypergeometric test was used to compute enrichment *P*-values (18).

## Results

### Innate Immunity Interactome Analysis Using InnateDB

In order to build, visualize, and analyze the DEG interaction networks in the context of innate immunity, we mapped our data to the InnateDB Network Analysis. Table 1 shows an example of InnateDB-curated molecular interactions generated from our data. In total, 2,458 InnateDB-curated interactions were generated from the 5,409 DEG at the acute stage of infection (FDR < 0.05). A smaller set comprising 224 InnateDB-curated interactions were generated from the 2,364 DEG at the chronic stage of infection (FDR < 0.05). The full molecular interactions from both DEG datasets are listed in Tables S1 and S2 in Supplementary Material, respectively.

**Table 1:** **Examples of InnateDB-curated molecular interactions generated from DEG demonstrated during the acute stage of ovine *F. hepatica* infection (week 1 postinfection)**.

Query Xref	Query name	Interactions	Full name	Interaction type	Tissue	Cell type	PMID^a^	Source database ID
ENSG00000141510	*TP53*	*TLR10:TP53*	*TLR10* interacts with *TP53*	Transcriptional regulation	Peripheral blood	T cell	21483755	IDB-224139
ENSG00000141510	*TP53*	*TLR6:TP53*	*TLR6* interacts with *TP53*	Transcriptional regulation	Peripheral blood	T cell	21483755	IDB-224137
ENSG00000184216	*IRAK1*	*NLRP12:IRAK1*	*NLRP12* physically interacts with *IRAK1*	Physical interaction	Peripheral blood, kidney cell line	Monocyte	16203735	IDB-113695; IDB-114001
ENSG00000184216	*IRAK1*	*ITGAM:IRAK1*	*ITGAM* physically interacts with *IRAK1*	Physical interaction	Peripheral blood, kidney cell line	Monocyte	11701612	IDB-113998; IDB-113999
ENSG00000141968	*VAV1*	*IL6ST:VAV1*	*IL6ST* interacts with *VAV1*	Physical association	Plasmacytoma cell line	B-cell	9013873	IDB-156849
ENSG00000136634	*IL10*	*IRF5:IL10*	*IRF5* interacts with *IL10*	Transcriptional regulation	Peripheral blood	Macrophage	21240265	IDB-223555

*^a^PMID refers to the publication that reported the interaction evidence that refers to the experimental procedures and conditions to support the interactions*.

Although curated interactions in InnateDB are annotated in a wide range of cell and tissue types, the majority of these interactions are from studies involving cell lines rather than primary cells (21). This is reflected in our results with 1,622 interactions from the acute stage and 159 interactions from the chronic stage of infection predicted from at least one source based on cell lines. Since the interactome is very much dependent on the context of a particular cell type under investigation (21), we focused on the interactions from studies involving PBMC-related cell types including B cells, T cells, NK cells, and monocytes. From samples representing the acute stage of infection, 458 PBMC-related interactions were identified from 2,458 InnateDB-curated interactions and 336 distinct DEG involved in these interactions (Table S1 in Supplementary Material). For the chronic stage of infection, 47 PBMC-related interactions were identified from 224 InnateDB-curated interactions, in which 55 distinct DEG were involved (Table S2 in Supplementary Material).

Among the 2,458 InnateDB-curated interactions representing the acute stage of infection, 92 interactions were identified, which were associated with evidence from fibrosis-associated cell types including fibroblast and stellate cell lines (the major cells involved in hepatic fibrosis) (Table S1 in Supplementary Material).

### Network Construction of Molecular Interactions Representing Acute/Chronic Stage of *F. hepatica* Infection

In order to gain a systems-level understanding of interactions of immunological interest, we used NetworkAnalyst to construct and visualize the molecular interaction networks (18). We first input the 336 DEG from the acute stage of infection that are involved in the PBMC-associated interactome with the corresponding fold-change values and generated a default network with 7,549 nodes and 24,354 edges (first-order interactors, including all identified proteins that directly interact with the input proteins, also known as seed nodes). It is notable that, apart from the seed nodes, there were a large number of directly interacting proteins identified, which made the default network too large and would lead to a “hairball” effect that rarely produces any informative outcome. For practical (visual, biological, and computational) reasons, a “Reduce” function was used to prune the network down to a more manageable size (200–2000 nodes) as recommended by Xia et al. (18), and a refined network with 331 nodes and 1,769 edges was retained for further analysis (zero-order interactions that refer to connections between two input seed nodes). The “Reduce” function retained only the seed nodes and the direct connections (edges) among them. Two widely used topological measures—“degree of connectivity” and “between centrality” were used to help identification of importance of nodes within the network (Figure S1 in Supplementary Material) (18). Degree of connectivity refers to the number of connections the node has to other nodes. Betweenness centrality refers to the number of shortest paths going through the node (22). In the current network (Figure S2 in Supplementary Material), the top five nodes with the highest node degree values are “reticuloendotheliosis viral oncogene homolog A” (RELA; degree value = 66), “tumor protein p53” (TP53; 65), “transcription factor Sp1” (SP1; 63), “amyloid precursor protein” (APP; 56), and “tyrosine 3-monooxygenase/tryptophan 5-monooxygenase activation protein” ζ (YWHAZ; 51). The top five nodes with the highest betweenness values are TP53 (betweenness value = 2,264.97), RELA (2,164.28), SP1 (1,505.93), “C-terminal Src kinase” (SRC; 1,271.48), and “breast cancer 1” (BRCA1; 1,269.96).

In the chronic stage of infection, 47 interactions are confirmed to stem from studies involving PBMC-related cell types, in which only 55 distinct DEG were involved. This figure was much lower than the recommended number of seed nodes (200–2,000) for network construction (18). Hence, we also performed network construction on the 186 distinct DEG involved in the 224 InnateDB-curated interactions using the same method for the dataset of the acute stage of infection. A preliminary network with 5,035 nodes and 11,422 edges was generated. After processing by the “Reduce” function built-in to NetworkAnalyst, a refined network with 158 nodes and 305 edges was retained for further analysis (only zero-order interactions among the input seed nodes were kept). Figure S3 in Supplementary Material shows the topology of the network generated. The top five nodes ranked by node degree are COPS5 (COP9 constitutive photomorphogenic homolog subunit 5), IKBKB (inhibitor of nuclear factor kappa-B kinase subunit β), YWHAE (14-3-3 protein epsilon, tyrosine 3-monooxygenase/tryptophan 5-monooxygenase activation protein epsilon), BRCA1, and JUN (Jun proto-oncogene, AP-1 transcription factor subunit) with their node degree values 21, 18, 17, 16, and 15 respectively. The top five nodes ranked by betweenness values are BRCA1, COPS5, LYL1 (lymphoblastic leukemia-associated hematopoiesis regulator 1), S-phase kinase-associated protein 2 (SKP2), and CSNK2B (Casein kinase 2, β polypeptide) with their betweenness values 271.25, 148.23, 139.17, 120.4, and 103.67, respectively.

### Function-First Approach Analysis

We first performed function-first approach analysis on the networks representing acute and chronic stages of infection using the Reactome database.

In total, 121 and 63 Reactome functional pathways were enriched (*P* < 0.001) in networks representing acute and chronic stages of infection, respectively. Tables 2 and 3 show the list of the top 25 pathways enriched in both stages of infection, respectively.

**Table 2:** **The top 25 enriched Reactome pathways within the network at the acute stage of infection**.

Pathway	Total	Hits	*P*-value
Immune system	1,140	114	1.62E−37
Innate immune system	521	69	1.00E−27
Toll-like receptors cascades	123	36	2.09E−26
Toll-like receptor 4 (TLR4) cascade	103	31	3.27E−23
Activated TLR4 signaling	100	29	3.03E−21
TRIF-mediated TLR3/TLR4 signaling	87	24	3.62E−17
MyD88-independent cascade	88	24	4.85E−17
Toll-like receptor 3 (TLR3) cascade	88	24	4.85E−17
MyD88:Mal cascade initiated on plasma membrane	81	23	8.42E−17
Toll-like receptor TLR1:TLR2 cascade	81	23	8.42E−17
Toll-like receptor TLR6:TLR2 cascade	81	23	8.42E−17
Toll-like receptor 2 (TLR2) cascade	81	23	8.42E−17
Toll-like receptor 10 (TLR10) cascade	74	20	2.74E−14
Toll-like receptor 5 (TLR5) cascade	74	20	2.74E−14
MyD88 cascade initiated on plasma membrane	74	20	2.74E−14
Cytokine signaling in immune system	286	37	2.78E−14
Signaling by interleukins	116	24	4.31E−14
Toll-like receptor 7/8 (TLR7/8) cascade	77	19	7.62E−13
MyD88 dependent cascade initiated on endosome	77	19	7.62E−13
Toll-like receptor 9 (TLR9) cascade	79	19	1.26E−12
TRAF6-mediated induction of NFkB and MAP kinases upon TLR7/8 or 9 activation	76	18	6.71E−12
Hemostasis	511	46	1.02E−11
Adaptive immune system	654	53	1.15E−11
TRAF6-mediated induction of proinflammatory cytokines	62	16	2.54E−11
Nucleotide-binding domain, leucine rich repeat containing receptor signaling pathways	55	15	4.58E−11

The pathways are ranked by their raw *P* values from functional enrichment analysis based on hypergeometric tests. (Note that multiple testing adjustments such as FDR are inappropriate here owing to the overlap and interdependence of genes within the pathways.)

**Table 3:** **The top 25 enriched Reactome pathways within the network at the chronic stage of ovine *F. hepatica* infection**.

Pathway	Total	Hits	*P*-value
MyD88:Mal cascade initiated on plasma membrane	81	13	5.45E−11
Toll-like receptor TLR1:TLR2 cascade	81	13	5.45E−11
Toll-like receptor TLR6:TLR2 cascade	81	13	5.45E−11
Toll-like receptor 2 (TLR2) cascade	81	13	5.45E−11
Activated TLR4 signaling	100	14	6.33E−11
Toll-like receptors cascades	123	15	9.39E−11
Toll-like receptor 4 (TLR4) cascade	103	14	9.53E−11
TRIF-mediated TLR3/TLR4 signaling	87	13	1.39E−10
MyD88-independent cascade	88	13	1.61E−10
Toll-like receptor 3 (TLR3) cascade	88	13	1.61E−10
Toll-like receptor 10 (TLR10) cascade	74	11	4.26E−09
Toll-like receptor 5 (TLR5) cascade	74	11	4.26E−09
MyD88 cascade initiated on plasma membrane	74	11	4.26E−09
Innate immune system	521	26	4.73E−09
TRAF6 (TNF receptor associated factor 6)-mediated induction of proinflammatory cytokines	62	10	9.96E−09
TRAF6-mediated induction of NFkB and MAP kinases upon TLR7/8 or 9 activation	76	10	7.54E−08
Toll-like receptor 7/8 (TLR7/8) cascade	77	10	8.57E−08
MyD88 dependent cascade initiated on endosome	77	10	8.57E−08
Toll-like receptor 9 (TLR9) cascade	79	10	1.10E−07
Immune system	1140	37	1.40E−07
TAK1 (also known as MAP3K7) activates NFkB by phosphorylation and activation of IKKs complex	22	6	3.87E−07
MAP kinase activation in TLR cascade	55	8	7.44E−07
TRAF6-mediated induction of TAK1 complex	16	5	1.82E−06
JNK (c-Jun kinases) phosphorylation and activation mediated by activated human TAK1	20	5	6.18E−06
Activation of the AP-1 family of transcription factors	10	4	6.95E−06

*The pathways are ranked by their raw P-values from functional enrichment analysis based on hypergeometric tests*.

**Figure 1:**
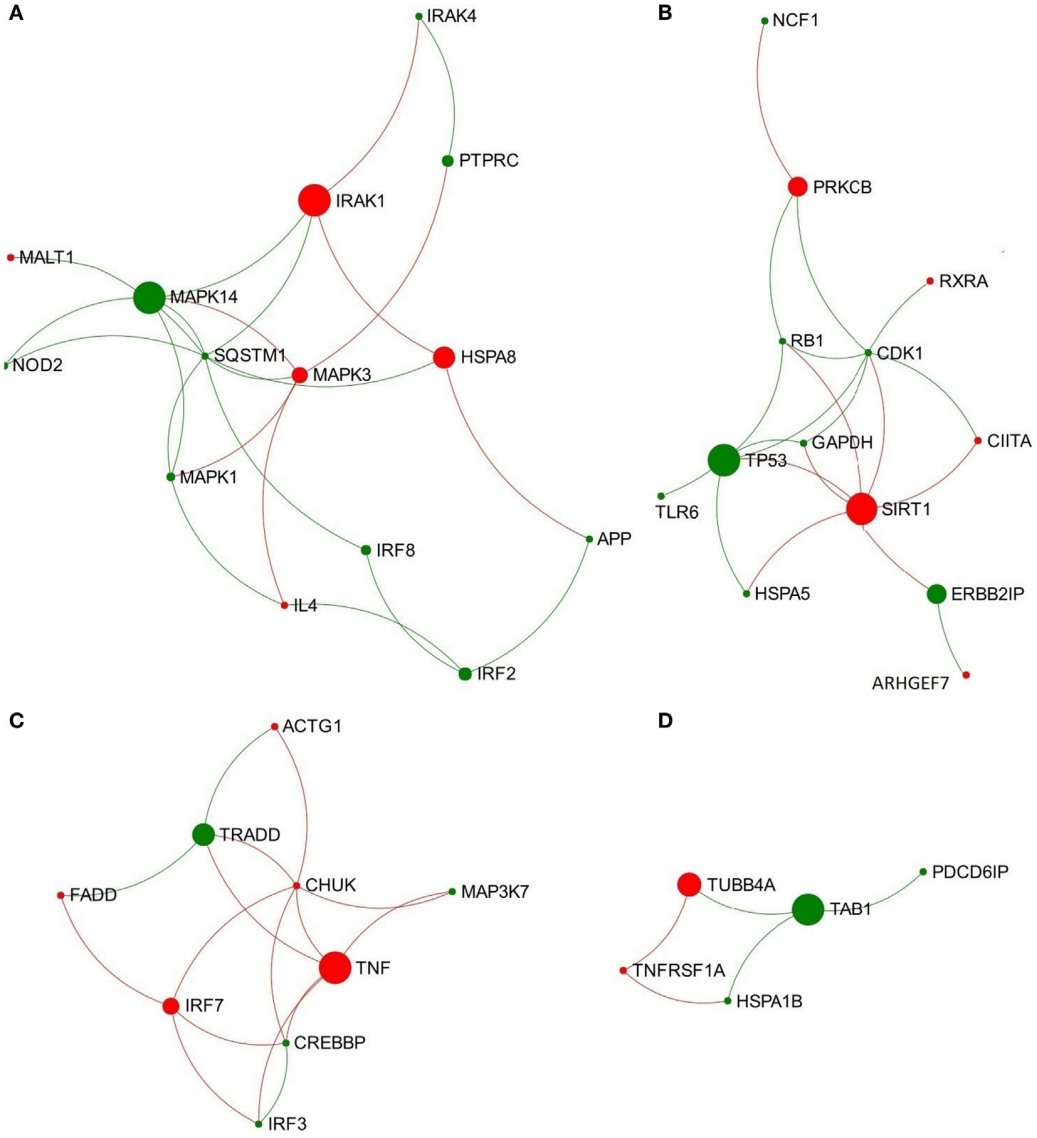
**Four significantly overlapped modules are extracted from the toll-like receptor 3/4-associated subnetwork from data representing acute stage of *Fasciola hepatica* infection**. **(A)** Module 1, **(B)** Module 2, **(C)** Module 3, and **(D)** Module 4. Red and green nodes represent genes showing increased and decreased expression, respectively. The size of nodes is proportional to their betweenness centrality values.

In the network representing the acute stage of infection, five pathways associated with TLR3/TLR4 signaling were ranked in the top 10 overlapping pathways identified, including “TLR4 cascade,” “activated TLR4 signaling,” “TRIF-mediated TLR3/TLR4 signaling,” “MyD88-independent cascade,” and “TLR3 cascade” (Table 2). Since TLR3 and TLR4 share a specific TRIF (TIR-domain-containing adapter-inducing interferon-β)-dependent pathway (unlike other TLRs) (23, 24), we extracted a subnetwork that contained all nodes involved in these five pathways (Table S3 in Supplementary Material). Subsequent module exploration analysis generated four significantly overlapping modules within this subnetwork (Figure 1). Then, functional enrichment analysis was repeated on these modules. Among them, Module 1 contains six nodes [NOD2, mitogen-activated protein kinase (MAPK) 14, MAPK1, MAPK3, interleukin-1 receptor-associated kinase (IRAK1), and IRAK4], which are involved in a range of TLR cascades, including TLR3/4/7/8/10, indicating a cross talk between TLR pathways. Module 3 contains five nodes mainly involved in My88-independent TRIF-dependent TLR signaling, including interferon regulatory factor 7 (IRF7), interferon regulatory factor 3 (IRF3), conserved helix-loop-helix ubiquitous kinase (CHUK), mitogen-activated protein kinase kinase kinase 7 (MAP3K7), and Fas-associated protein with death domain (FADD).

**Figure 2:**
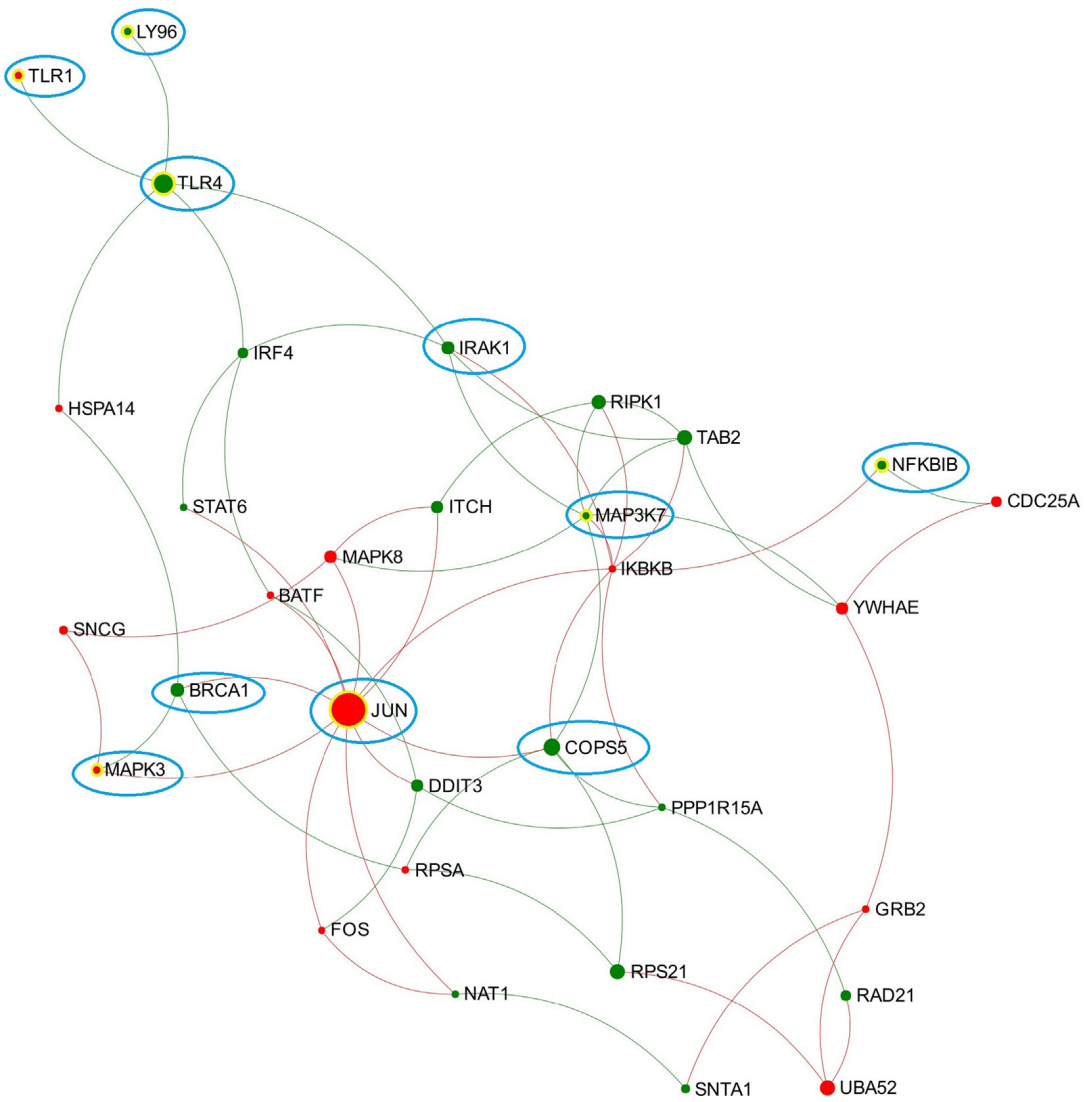
**Expression patterns of the toll-like receptor (TLR) 3/4-associated subnetwork extracted from data representing chronic stage of *Fasciola hepatica* infection**. Red and green nodes represent genes showing increased and decreased expression, respectively. The size of nodes is proportional to their betweenness centrality values. Several nodes that consistently presented in the TLR3/TLR4-associated subnetworks (generated from acute and chronic stages of infection, respectively) are highlighted with blue cycle.

Similar to the findings for the acute stage of infection, the five pathways related to TLR4/TLR3 also presented in the top 10 enriched pathways in the chronic stage of infection, including “TLR4 cascade,” “activated TLR4 signaling,” “TRIF-mediated TLR3/TLR4 signaling,” “MyD88-independent cascade,” and “TLR3 cascade.” We extracted a subnetwork that contained all nodes that are involved in these five pathways (Table S3 in Supplementary Material; Figure 2). There were no significant overlapping modules found within this subnetwork, which may be due to the small size of the subnetwork.

### Connection-First Approach Analysis

Almost all the top ranked pathways enriched by the function-first approach in both networks are associated with immune responses. It is, thus, clear that the pre-enrichment of DEG from the immune-related interactome (which have been confirmed in the context of PBMC-related cell types) is a reliable and effective way to extract immune-related networks and refine the network size for further analysis. In order to obtain tightly clustered modules, we conducted a complementary approach (connection-first) to re-analyze the two networks separately.

#### Connection-First Approach Analysis on Networks Representing the Acute Stage of *F. hepatica* Infection

**Figure 3:**
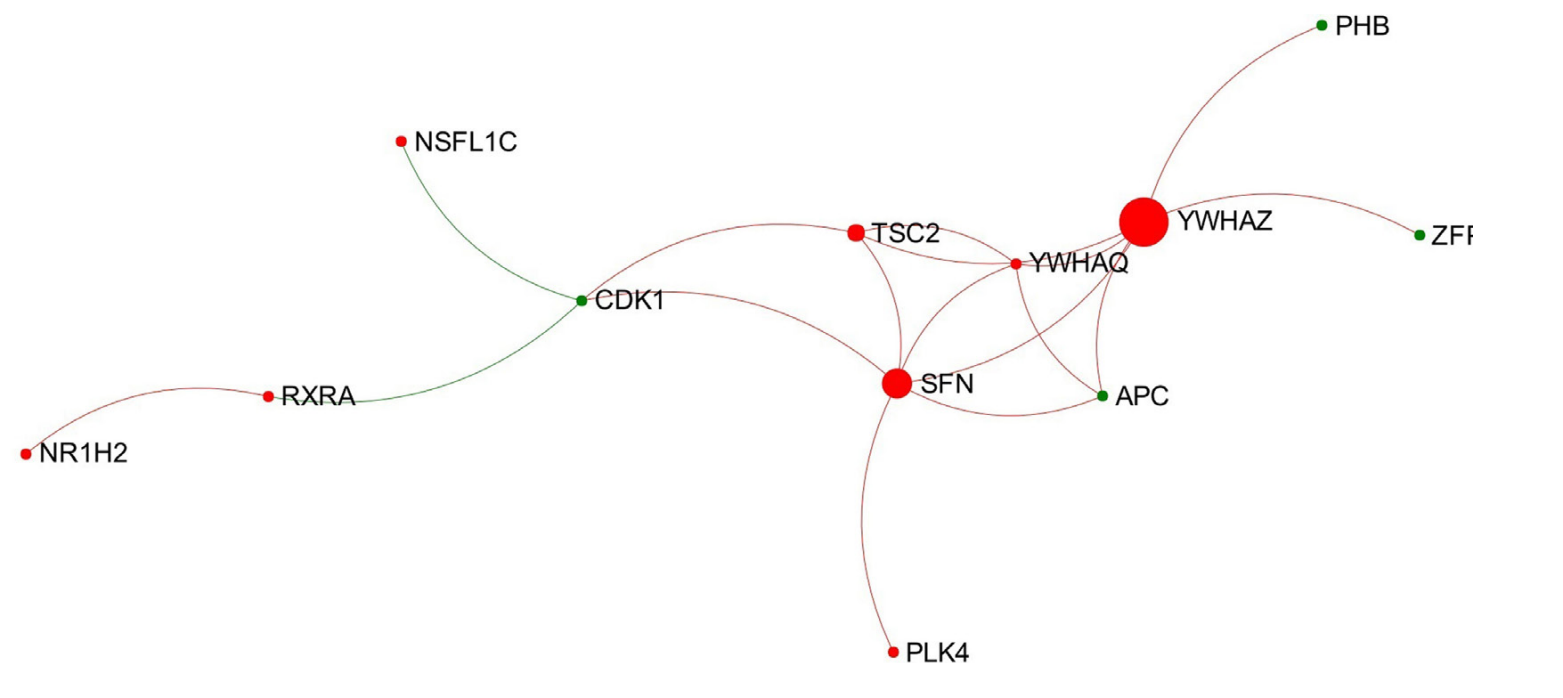
**Expression pattern of Module 6 extracted from the network representing acute stage of *Fasciola hepatica* infection**. Red and green nodes represent genes showing increased and decreased expression, respectively. The size of nodes is proportional to their betweenness centrality values. The sequence number is ranked by module size.

In total, 18 individual modules were generated from the network representing the acute stage of infection. Among them there were nine modules that showed module significance (*P* < 0.05). The expression patterns of these modules are shown in Figure S4 in Supplementary Material. Some of the modules showed general trends in expression pattern. For instance, Modules 2, 3, and 7 were likely to show decreased expression, while Module 6 was likely to show increased expression (Figure 3).

Then, we conducted function enrichment analysis on each module mainly based on Reactome databases [in some cases, if only a very low ratio of nodes within a module hit any Reactome pathways, KEGG, or GO-Biological Process (GO-BP) terms were alternatively used to interpret the module]. Only the pathways/functions with *P*-value <0.001 were retained for further analysis.

Modules 1 and 2, which contain the most nodes (52 and 44, respectively), are mainly associated with immune-system pathways. The most significant Reactome pathways enriched in Module 1 are “Immune System” (16 hits) and “Cytokine signaling in immune system” (8 hits). The most significant Reactome pathway enriched in Module 2 is “Immune System” (24 hits), followed by the pathway “Innate immune system” (16 hits). Module 4, which contains 18 nodes, is similar to Module 2, in which the most significant pathways are also “Immune system” (12 hits) and “Innate immune system” (9 hits). These results indicate that the three top-ranked modules are likely to perform independent functions in the immune system during *F. hepatica* infection. In general, this result is consistent with the result of the function-first approach, in that the top two enriched pathways are “Immune system” and “Innate immune system.”

The most significant Reactome pathways enriched in Module 3 (containing 22 nodes) are all associated with TLR signaling, such as “Activated TLR4 signaling” (eight hits), “TLR 4 cascade” (eight hits), “TLRs cascades” (eight hits), “TLR 7/8 cascade” (seven hits), and “MyD88-independent cascade” (seven hits), etc. There are eight key nodes (DEG) from Module 3 consistently enriched in these pathways, including NOD2, MAPK14, IRAK1, IRF7, FADD, activating transcription factor 2 (ATF2), MAP3K1, and NF-kappa-B inhibitor β (NFKBIB). It is thus possible that Module 3 plays a fundamental role in regulation of various TLR signaling/cascades during *F. hepatica* infection.

Module 6 (containing 12 nodes) was the only one to show an obvious increased expression pattern among the nine modules (Figure 3). Further functional enrichment revealed that the top four Reactome pathways enriched in this module are all involved in apoptosis, particularly in the intrinsic apoptosis pathway/mitochondrial pathway, including “activation of BAD and translocation to mitochondria,” “activation of BH3-only proteins,” “intrinsic pathway for apoptosis,” and “apoptosis.”

Functional analysis based on Reactome, KEGG, and GO-BP suggests that Module 7 (containing 11 nodes) is related to cytokine signaling. There are two nodes [colony-stimulating factor 2 receptor β (CSF2RB) and colony-stimulating factor 2 (CSF2)] that hit the Reactome pathways “interleukin receptor SHC signaling,” “IL2 signaling,” and “IL3, IL5, and GM-CSF signaling.” Four nodes [CSF2RB, CSF2, IL10, and chemokine (C-X3-C motif) receptor 1 (CX3CR1)] hit the KEGG pathway “cytokine–cytokine receptor interaction.” Five nodes [IL10, NFATC2 (nuclear factor of activated T-cells, cytoplasmic, calcineurin-dependent 2), FOXP3, CSF2, and TIA1 (TIA1 cytotoxic granule-associated RNA binding protein)] hit the GO-BP term “Cytokine production.”

There were a very low number of nodes within Module 5, 8, and 9 associated with any pathways of either Reactome or KEGG. However, the majority of nodes from Module 5 (nine nodes, 69%) significantly hit the GO-BP term “defense response.” Five nodes (55%) from Module 8 are involved in the GO-BP term “regulation of cytokine production.” All five nodes within Module 9 significantly hit the GO-BP term “regulation of protein metabolic process.”

#### Connection-First Approach Analysis on Networks Representing the Chronic Stage of *F. hepatica* Infection

In total, 11 individual modules were generated from the network representing the chronic stage of infection. Among them there were four modules with significance (*P* < 0.05). The expression patterns and the node labels of the four modules are shown in Figure S5 in Supplementary Material.

**Figure 4:**
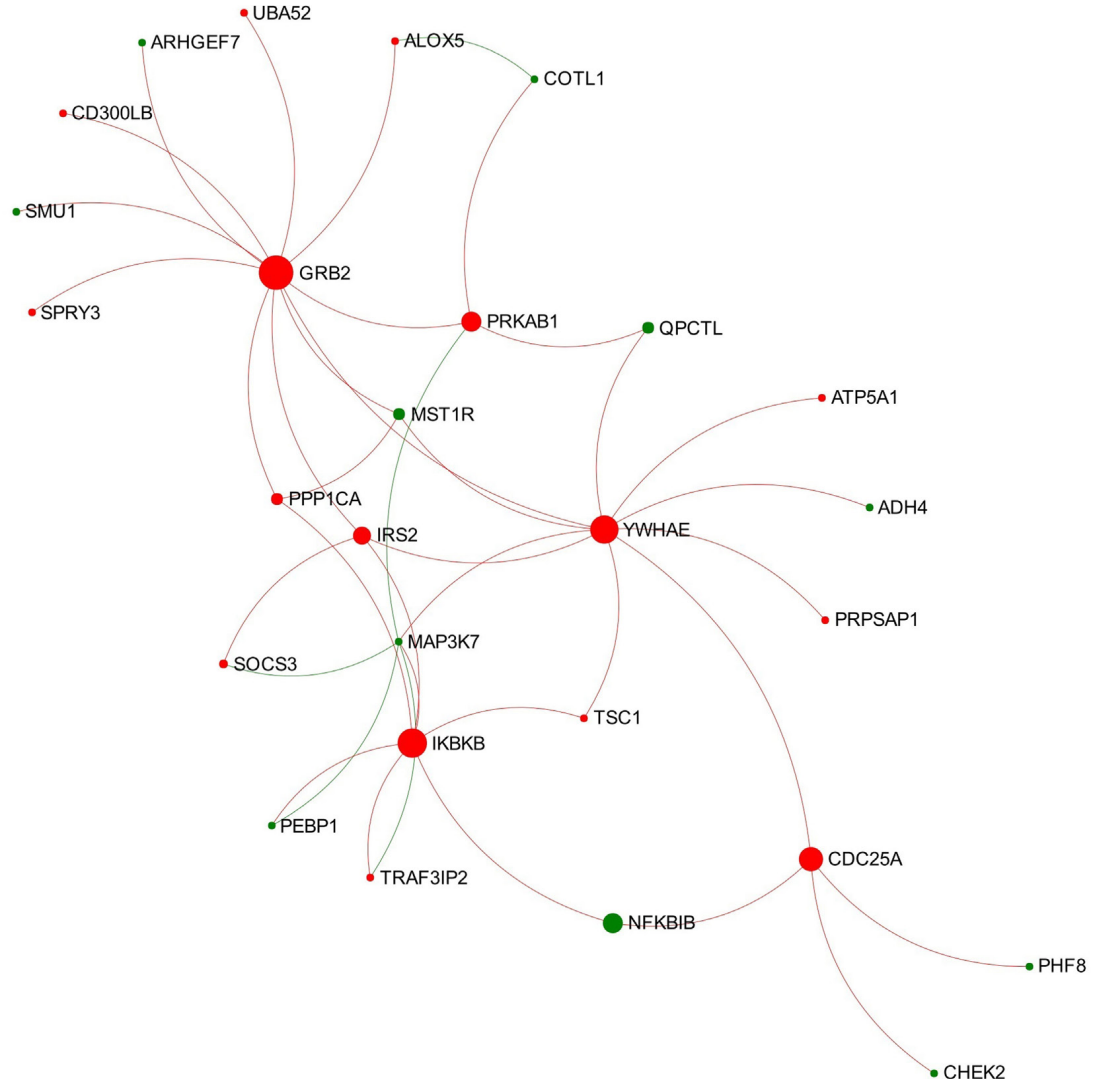
**Expression pattern of Module 1 extracted from the network representing chronic stage of *Fasciola hepatica* infection**. Red and green nodes represent genes showing increased and decreased expression, respectively. The size of nodes is proportional to their betweenness centrality values.

Module 1 appears to be activated since most of the involved nodes showed increased expression in the chronic stage of infection (Figure 4). Further function enrichment analysis showed that three B-cell receptor (BCR) related pathways were significantly enriched (ranked top 3 functions) in this module including “TAK1 activates NFkB by phosphorylation and activation of IKKs complex,” “Downstream signaling events of B-cell receptor,” and “Signaling by the B-cell receptor.”

Functional analysis showed that there were no dominant pathways enriched in Modules 2, 3, and 4 neither in Reactome nor in KEGG. However, there were several GO-BP terms associated with lymphocyte activation significantly enriched in Module 2 (*P* < 0.001), and five nodes within Module 2 were involved in those terms, including interleukin-20 receptor β chain (IL20RB), STAT6, IFNG, IRF4, and TNFRSF13B. This indicates that Module 2 may be involved in the regulation of lymphocyte activation during the chronic stage of *F. hepatica* infection.

## Discussion

**Figure 5:**
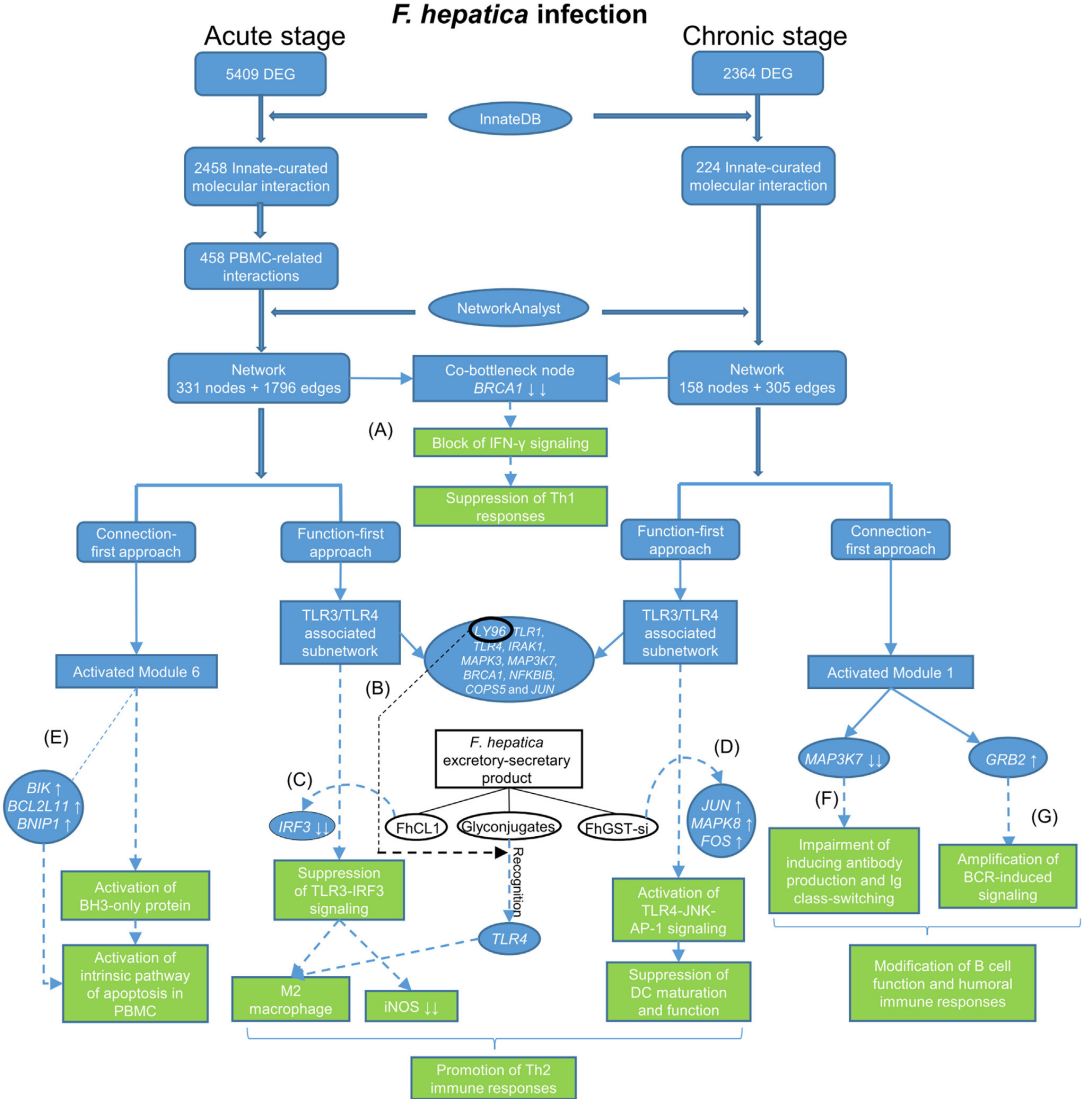
**Flow diagram showing data process and how the main findings lead to a better understanding of immune responses to *Fasciola hepatica* infection**. The blue tabs and solid arrows refer to the pipeline of data mining and main results. The green tabs and dotted arrows refer to hypothesis generation: **(A)** *F. hepatica* infection may block IFN-γ signaling through consistently suppressing the transcript expression of breast cancer 1. **(B)** LY96 may play a role in recognition of fluke-derived glycan residues by Toll-like receptor 4 (TLR4) in macrophages. **(C)** *F. hepatica* cathepsin L1 (FhCL1) induces M2 macrophage phenotypes through blocking TLR3-IRF3 pathway, possibly by decreasing transcript expression of IRF3. **(D)** *F. hepatica* sigma class glutathione transferase (FhGST-si) suppresses dendritic cells maturation through activating TLR4-JNK-AP-1 pathway, possibly by increasing transcript expression of JUN, MAPK8, and FOS. **(E)** *F. hepatica* may induce apoptosis of peripheral blood mononuclear cells through intrinsic pathway, possibly by increasing transcript expression of BIK, BCL2L11, and BNIP1. **(F)** *F. hepatica* infection may modulate humoral immune responses through regulating transcript expression of MAP3K7. **(G)** *F. hepatica* infection may modulate B-cell receptor signaling through activating growth factor receptor-bound protein 2 transcript expression.

In this study, we re-analyzed our previous RNA-seq data to generate a refined molecular interactome in the context of innate immune responses to *F. hepatica* infection in sheep. To visualize this interactome, we constructed networks based on the relevant DEG by using NetworkAnalyst and employed two heuristic approaches (function-first and connection-first) to interpret the networks (Figure 5). For the acute stage of infection, the results from the two approaches were partially overlapping. For instance, the top two pathways highlighted in the function-first approach were “Immune system” and “Innate Immune System,” whereas the two largest modules observed in the connection-first approach were also related to the general function, “Immune System” and “Innate Immune System.” In addition, TLR-related pathways were top-ranked in the function-first approach. This was consistent with the third largest module (Module 3) generated from the connection-first approach that mainly enriched TLR signaling. However, the two approaches also enriched different functions, for example, “Signaling by Interleukins” in the function-first approach and “Activation of BH3-only proteins” in Module 6 from the connection-first approach. In the dataset from the chronic stage of infection, the top functions observed in the function-first approach were TLR-associated pathways. However, there was no module from the connection-first approach related to TLR signaling. These inconsistencies, resulting from the different priorities for function and connectivity, respectively, may reflect the inherent biases of each approach. However, investigation of the network from different angles provides us with a more comprehensive interpretation of the whole network and a better understanding of the innate immune response to *F. hepatica* infection.

### Fibrosis-Associated Interactions in the Acute Stage of *F. hepatica* Infection

Ninety-two InnateDB-curated interactions from the acute stage of infection were identified, which included evidence from fibrosis-associated cell types including fibroblast and stellate cell lines. Some genes related to the TGFβ-mediated fibrogenic progresses (such as SMAD3, SMAD4, COL1A1, and TGFB1) were involved in the 92 interactions. In our previous IPA analysis on this dataset, we hypothesized that the increased expression of these genes may be possibly associated with the fibrosis in the ovine liver during *F. hepatica* infection (15). A previous study using hepatic lymph nodes has also proposed that TGFβ1 plays a central role in fibrosis during ovine *F. hepatica* infection (25). It is well known generally that persistent TGFβ signaling leads to excessive fibrosis and ultimately scarring of internal organs (26). TGFβ induces the transcription of the gene COL1A1, encoding collagen type I, which is the major fibrous collagen and plays a central role in wound-healing (26). Another study demonstrated that the transcript expression levels of TGFB1 and COL1A1 were significantly increased in ovine liver tissue at eight wpi of *F. hepatica* (27). Notably, the cells being analyzed in this study are PBMC, and it is, therefore, notable that 92 interactions also have been demonstrated in fibrosis-associated cell types that may directly interact with *F. hepatica* during the local infection in the liver. These data may indicate that in response to *F. hepatica* infection, blood mononuclear cells could migrate into the infected liver parenchyma and release TGFB1 and COLIA1 to activate fibrosis-associated cell types (e.g., stellate cells) and facilitate hepatic fibrosis. Or perhaps the ovine PBMC and fibrosis-associated cell types might perform same gene expression changes associated with TGFβ—collagen pathway in the context of *F. hepatica* infection. Further investigations into links between the systemic immune system and local infection in the liver will be valuable.

### Hub and Bottleneck Molecules Play Potential Roles in Immune Regulation during *F. hepatica* Infection

Node importance in network analysis can be estimated as degree of connectivity (the number of connections the node has to other nodes) and betweenness centrality (the number of shortest paths going through the node) (22). Nodes with a high degree of centrality are referred to as “hub nodes,” whereas nodes with a high betweenness centrality are referred as “bottleneck nodes” (28). Notably, in our analysis, some molecules performed as both hub and bottleneck nodes in the network, indicating their crucial roles in regulating the innate immune response to *F. hepatica* infection. For instance, RELA and SP1 are in the top of both hub and bottleneck nodes in the network representing the acute stage of infection. RELA encodes the transcription factor NF-kBp65, one of the active subunits of the NF-Kb complex involved in NF-Kb binding to DNA. Previous studies have suggested that NF-kBp65 participates in adaptive immunity and responses to invading pathogens *via* NF-kB activation (29). One study has demonstrated that *F. hepatica* tegument can target NF-kBp65 and suppress its protein expression in DC, which subsequently resulted in decreased production of pro-inflammatory cytokines in DC (14). It has also been shown that the recombinant form of a major *F. hepatica*-secreted molecule, sigma class glutathione transferase (rFhGST-si) could induce partial maturation of DC *in vitro* by enhancing the protein production of NF-kBp65 and signaling through it (12). These studies suggest that different components of *F. hepatica* can affect DC maturation and function through regulating *RELA* expression in different ways. *SP1* is a zinc finger transcription factor that is a central mediator of IL-10 induction (30, 31). Previous studies proposed that a helminth immune modulator (cystatin from *Acanthocheilonema viteae*) can bind to macrophages and activate SP1 *via* p38 signaling pathway to initiate transcript expression of IL10 (32). Since IL10 is an important regulatory cytokine induced by *F. hepatica* infection (33), the high importance of SP1 within the network may indicate its potential role in regulating IL10 during the acute stage of *F. hepatica* infection.

Breast cancer 1 is one of the top five bottleneck nodes in both networks at the acute and chronic stages of infection. Much current research on BRCA1 focuses on its role in DNA damage repair (34), but its role in the context of immunology/infection is rarely characterized. One study suggested that BRCA1 can activate IFN-γ signaling to induce target gene IRF-7 expression and to subsequently stimulate the innate immune response and elicit apoptosis (35). In our DEG data, BRCA1 showed a significantly decreased transcript expression at the acute stage of *F. hepatica* infection (Table S1 in Supplementary Material), which dropped down to an even lower level at the chronic stage of infection (Table S2 in Supplementary Material). This provides a potential mechanism for the decreased transcription of IFNG in our DEG dataset (Table S1 in Supplementary Material) and for the downregulation of Th1 immune responsiveness seen in *F. hepatica* infection. The high betweenness value of BRCA1 in both networks indicates that *F. hepatica* infection may block IFN-γ signaling and further attenuate Th1 immune responses through suppressing the expression of BRCA1.

### The Function-First Approach Revealed New Features of TLR3/TLR4 Signaling in the Context of *F. hepatica* Infection

The initiation of TLR3/TLR4 signaling by *F. hepatica* infection and its downstream effects are not fully understood. The ligand of TLR3 is double-stranded RNA (dsRNA), which has been well-studied in the field of viral infection. One study has demonstrated that dsRNA from *Schistosoma mansoni* eggs is a natural ligand for TLR3, and this egg-derived dsRNA was capable of activating DC through a fully TLR3-dependent pathway (36). Hence, regulation of TLR3-dependent cellular pathways could be used by helminth parasites to modulate host innate immune responses. TLR3 suppression has been demonstrated to play a role in promoting Th2 immune responses to *S. mansoni* infection. In TLR3 gene-deficient mice, the absence of TLR3 resulted in significantly decreased Th1-associated factors (e.g., IL12) and significantly increased Th2 cytokines and chemokines in response to *S. mansoni* infection (37). In addition, macrophages from TLR3 deficient mice were alternatively activated (37). These data suggest that the absence of TLR3 leads to a strongly skewed Th2 response during *S. mansoni* infection. During the early stage of infection, the penetration and migration of *F. hepatica* can cause severe injury of liver tissue. To avoid massive immunopathology, *F. hepatica* migrating juveniles have to prevent cells of the innate immune system promoting a Th1-adaptive response, thus making them more likely to drive the development of Th2 immune responses (38). Regulation of TLR3 signaling has been considered to be involved in this polarization process. Previous studies have demonstrated that *F. hepatica* cathepsin L1 (FhCL1, a major component of the excretory-secretory proteins) could suppress the production of proinflammatory mediators from LPS-stimulated macrophages by inhibiting TLR3 signaling (38). In this study, Module 3 extracted from the TLR3/TLR4-associated subnetwork at the acute stage of infection contains five nodes that play crucial roles in My88-independent TRIF-dependent TLR signaling (Figure 1). Among them, IRF3 is an important transcription factor involved in the downstream regulation of TLR3-TRIF (39), which regulates type 1 IFNs (e.g., IFN-β), leading to subsequent induction of iNOS (inducible nitric oxide synthase, encoded by gene NOS2) expression (38, 39). IRF3 showed significantly decreased expression at the acute stage of infection (logFC = −7.07) (Table S1 in Supplementary Material), with similarly low expression levels until the chronic stage of infection (15). The decreased expression of IRF3 and the related Module 3 might bridge the suppression of TRIF-dependent TLR3 signaling to the extremely decreased transcript expression of NOS2 during *F. hepatica* infection observed in our previous study (15). iNOS catalyzes the conversion of arginine into citrulline and nitric oxide (NO), which is a defense mechanism in response to pathogen invasion (40). The suppression of NOS2 transcript expression in macrophages is an important characteristic of *F. hepatica* infection (41), which could be one mechanism of immune evasion by the parasite to avoid damage from host-generated nitric oxide. Previous studies have shown that FhCL1 can suppress murine macrophage activation by the degradation of TLR3 protein within endosomes, rather than downregulation of TRIF and TLR3 transcript expression (38). This is consistent with the fact that TRIF and TLR3 are absent in our DEG datasets. It would be useful to investigate the effect of FhCL1 on downstream TLR3 signaling, such as transcript expression of IRF3. Our study also highlighted an interaction between TLR3 and TLR4 TRIF-dependent pathways, which may be affected by FhCL1 (38). However, other as yet unidentified mechanisms may be involved in the blocking of TRIF-dependent TLR3/TLR4 pathways induced by *F. hepatica* at the transcription regulation level.

Toll-like receptor 4 is well known as the receptor for bacterial endotoxins, several viral proteins, and polysaccharide and plays a pivotal role in the induction of inflammatory responses (42). In comparison to TLR3, there are more studies on the association between TLR4 and *F. hepatica* infection. One previous study suggested that FhES was capable of inhibiting the activation of bovine macrophages by TLR4 antagonists, indicating *F. hepatica* may modulate the host innate immune response through either altering the expression of TLR4 or interfering with its subsequent signaling (43). A recent study suggested that TLR4 may be involved in *F. hepatica* fatty acid-binding protein (FhFABP) induced alternative-activation of human macrophages (44). Another study suggested that FhFABP can inhibit TLR4 activation in macrophages and suppresses the inflammatory cytokines induced following incubation of LPS (45). It has also been suggested that TLR4 plays a crucial role in mediating the activation of a suppressive DC phenotype by two major components of FhES, FhCL1 and FhGST-si (12). However, TLR4 does not always account for the modulation of host DC. One study has demonstrated that FhTeg suppresses DC maturation and function independently of the TLR4 pathway, since it still functioned in DC generated from TLR4-deficient mice (14). Hence, it can be concluded that different components of *F. hepatica* have diverse influence on TLR4 transcript expression and related signaling, and TLR4 may play various roles in different cell types during *F. hepatica* infection. In the TLR3/TLR4-associated subnetwork from the chronic stage of infection (Figure 2), JUN has the highest node degree and betweenness values. JUN encodes the transcription factor c-Jun, which can be activated and phosphorylated by c-Jun N-terminal kinases (JNK) (46). Previous studies suggested that JNK was implicated in the TLR4-mediated intracellular signaling pathway through which rFhGST-si (the recombinant form of one major *F. hepatica* secreted protein) signaled to suppress DC maturation and function (12). Downstream of the JNK signaling pathway, activated c-Jun, and c-Fos function as subunits of a group of dimeric transcription factors were collectively referred to as activating protein (AP-1) that regulate transcript expression of target genes (47). It has been proposed that AP-1 complexes containing c-Fos might repress genes that are implicated in DC maturation, since continued expression of c-Fos was detrimental for DC maturation (48). In our analysis, genes coding JNK (MAPK8), c-Jun (JUN), and c-Fos (FOS) were all implicated in the subnetwork from the chronic stage of infection (Figure 2), and all showed significantly increased transcript expression levels at this stage (Table S2 in Supplementary Material). Hence, we hypothesize that activation of the TLR4-JNK-AP-1 signaling pathway could be a potential mechanism used by *F. hepatica* to modulate maturation and function of host DC during chronic infection.

By comparing the TLR3/TLR4-related subnetworks generated from acute and chronic stages of infection, we found several nodes (including LY96, TLR1, TLR4, IRAK1, MAPK3, MAP3K7, BRCA1, NFKBIB, COPS5, and JUN) that were consistently involved in both subnetworks (Figure 2), suggesting that these DEG may play a consistent role in regulation of TLR3/TLR4 function during *F. hepatica* infection. Among them, LY96 encodes a protein named lymphocyte antigen 96 (also known as MD2), which has been shown to play a role in the binding between LPS and TLR4 (49). Our group previously proposed that the ability of FhES to produce M2 macrophages may be partially dependent on parasite glycan residues, and the interaction between FhES and TLR4 is possibly involved in this mechanism (43). Given the consistent expression regulation of LY96 in this study and its role in TLR4 function, we propose that LY96 is critical in TLR4 recognition of fluke-derived glycan residues and may play a role in induction of M2 macrophages in the presence of *F. hepatica* secretory glycoproteins.

In summary, we provide here the first investigation of gene networks associated with TLR3/TLR4 signaling/cascade within PBMC during *F. hepatica* infection, which provides useful information for further investigating the roles of TLR3/TLR4 in single cell types, during helminth infection.

### The Connection-First Approach Revealed a Novel Module Implicated in Intrinsic Apoptosis in Early Stage of *F. hepatica* Infection

Module 6 extracted from the network representing the acute stage of infection significantly enriched four Reactome pathways associated with intrinsic apoptosis and shows a general trend of increased gene expression (Figure 3). This may indicate an activated intrinsic pathway of apoptosis in PBMC at early stage of *F. hepatica* infection, which is in agreement with our previous observations from the IPA pathway “Death Receptor Signaling” and “Apoptosis Signaling” (15). BH3-only proteins, structurally characterized by the BH3 domain [Bcl-2 homology (BH) domain 3], are a main pro-apoptotic subgroup in the BCL-2 (B-cell CLL/lymphoma 2) family that are crucial regulators of apoptosis during the intrinsic pathway (50). To date, several BH3-only proteins have been discovered in human and mouse (51). In our DEG data, there were three BH3-only member genes showing significantly increased expression during the acute stage of infection, including BIK (BCL-2-interacting killer; also known as NBK; logFC = 5.04), BNIP1 (BCL2/adenovirus E1B 19 kDa interacting protein 1; logFC = 1.69), and BCL2L11 (Bcl-2-like protein 11; also known as BIM; logFC = 1.43). In addition, one gene encoding another BH3-only protein, PMAIP1 (phorbol-12-myristate-13-acetate-induced protein 1; also known as NOXA; logFC = 3.89), also showed an increased expression in chronic stage of infection (15). Previous studies have shown that BIM is likely to be involved in apoptosis of lymphocytes [summarized from Strasser (50)], and also has a crucial role in the termination of T-cell immune responses (52, 53). We hypothesize that these four BH3-only proteins may be associated with previously observed apoptosis of peripheral blood immune cells during *F. hepatica* infection (54, 55). Since the top three enriched pathways in Module 6 were associated with BH3-only proteins, the increased expression of these BH3-only genes in the DEG dataset could be potentially attributed to the activation of Module 6.

### The Connection-First Approach Uncovered a BCR-Associated Module in the Chronic Stage of *F. hepatica* Infection

Module 1 extracted from the network representing the chronic stage of infection shows a general trend of activation and enriched three BCR-related pathways (Figure 4). Several nodes involved in all the three pathways were UBA52 (ubiquitin A-52 residue ribosomal protein fusion product 1), growth factor receptor-bound protein 2 (GRB2), IRS2 (insulin receptor substrate 2), MAP3K7 (also known as TAK1), IKBKB, and NFKBIB. BCR plays a critical role in initiation of B-cell responses and guides cell maturation, survival, energy, and the production of antibodies in plasma B-cells (summarized from Dal Porto et al. (56) and Liu et al. (57). As shown in Figure 4, GRB2 is the most important node in this module, which has the highest degree and betweenness values. GRB2 encodes an adapter protein that plays a role in recruiting and assembling the BCR signalosome and the amplification of its signal (58, 59). In another RNA-seq study on ovine PBMC following *F. hepatica* infection, the authors suggested that *F. hepatica* infection might induce activation of BCR through downregulating CD22, a negative modulator of BCR (60). Although CD22 was not detected in our DEG dataset, our data do suggest that the increased transcript expression of GRB2 and the associated module might be involved in activation of downstream signaling of BCR during *F. hepatica* infection. Another highlighted DEG of interest is MAP3K7, encoding an enzyme that is critical for B-cell maturation and BCR-mediated B-cell proliferation (61, 62). It has previously been demonstrated that mice with B-cell-specific MAP3K7 deficiency have a significantly reduced basal titers of IgG1, IgG2a/b, IgG3, and IgA in serum compared with those of control mice (61). In addition, the production of antigen-specific IgM and IgG1 in response to a T cell-dependent antigen (nitrophenol-chicken g-globulin) was considerably impaired in MAP3K7-deficient mice compared with that of controls. This study indicated that MAP3K7 is indispensable for *in vivo* induction of humoral immune responses as well as isotype switching (61). Notably, transcript expression levels of MAP3K7 in our study consistently decreased from the acute through to the chronic stage of *F. hepatica* infection (Tables S1 and S2 in Supplementary Material). It is well known that *F. hepatica* influences IgG1/IgG2 class-switching and antibody affinity maturation (63–65). Hence, we propose that the consistent suppression of MAP3K7 and its role in this module could be implicated in the *F. hepatica*-induced modulation of BCR signaling and humoral immune responses.

### Summary Findings

This study for the first time investigates the global gene expression changes associated with innate immunity to *F. hepatica* infection at a systems level. Our findings revealed novel insights into *F. hepatica*-mediated modulation of host immune responses, which facilitate parasite survival and chronic infection. The data from this study will benefit the rational design of vaccines which to be effective, will need to overcome the extreme parasite-mediated immunoregulation seen in fasciolosis. As well as their direct applicability in the design of vaccines for the control of this major disease of livestock, these insights also contribute to the general understanding of helminth-mediated immunoregulation [reviewed in Ref. (66)]. A substantial body of evidence now points to the multiple layers of immunoregulation and immunodiversion employed by helminths to limit immunopathology and survive long-term in their hosts. However, a complete mechanistic understanding of how such profound effects of the host’s immune system are orchestrated is still developing. Furthering this understanding is important not only for advancing helminth vaccinology, but also for harnessing similar immunoregulatory strategies for the treatment of autoimmune and immunopathological states. Our data were obtained using a complex PBMC population, and it is, therefore, not surprising that a complex pattern of gene expression changes was observed. Further verification of specific pathway changes in individual cell types is an obvious next step.

## Author Contributions

YF, JB, and GM conceived and designed the experiments. YF performed the experiments and analyzed the data. YF and GM contributed to the writing of the manuscript. GM, KK, and JB contributed to the writing and editing of the manuscript critically for important intellectual content.

## Conflict of Interest Statement

The authors declare that the research was conducted in the absence of any commercial or financial relationships that could be construed as a potential conflict of interest.

## Supplementary Material

Figure S1 **Network schematic**. The network is presented as an undirected graph with nodes as protein/DEG (circle) and edges (line) indicating interactions between two connecting nodes. Node importance is estimated as degree centrality (the number of connections the node has to other nodes) and betweenness centrality (the number of shortest paths going through the node). Green represents nodes with both high degree and betweenness values. Blue represents nodes with a low degree but high betweenness value. Yellow represents nodes with a high degree but low betweenness value. Red represents nodes with a low degree and betweenness value.

Figure S2 **Topology of the peripheral blood mononuclear cell- associated Interactome (336 DEG, acute stage of infection)**. The network is presented as an undirected graph with nodes as protein/DEG (green arrows) and edges (blue arrows) indicating interactions between two connecting proteins. Node importance was estimated as degree centrality (the number of connections the node has to other nodes) and betweenness centrality (the number of shortest paths going through the node). Nodes with higher degree and betweenness values are darker red and larger, respectively. The top five nodes with the highest degree or betweenness values are highlighted with blue circles.

Figure S3 **A topologic view of the network (186 DEG, Chronic stage of infection)**. Red and green nodes represent genes showing increased and decreased expression, respectively. The top five hub nodes with highest degree or betweenness values are highlighted with blue circles.

Figure S4 **Expression pattern of nine modules extracted from the network representing acute stage of *Fasciola hepatica* infection**. Red and green nodes represent genes showing increased/decreased expression, respectively. The size of nodes is proportional to their betweenness centrality values. The sequence number is ranked by module size.

Figure S5 **Expression patterns of four modules extracted from the network representing chronic stage of *Fasciola hepatica* infection**. Red nodes are upregulated and green nodes are downregulated. The size of nodes is proportional to their betweenness centrality values.

Table S1 **InnateDB-curated molecular interactions and associated DEG from acute stage of *Fasciola hepatica* infection**.

Table S2 **InnateDB-curated molecular interactions and associated DEG from chronic stage of *Fasciola hepatica* infection**.

Table S3 **DEG information of TLR3/TLR4 associated subnetwork extracted from network of acute and chronic stages of *Fasciola hepatica* infection**.
