# Supplementary figures and images for: Network Analysis of the Systemic Response to Fasciola hepatica Infection in Sheep Reveals Changes in Fibrosis, Apoptosis, Toll-Like Receptors 3/4, and B Cell Function
Source: Front Immunol. 2017 Apr 25;8:485. doi: 10.3389/fimmu.2017.00485 (PMC5403899; doi:10.3389/fimmu.2017.00485)

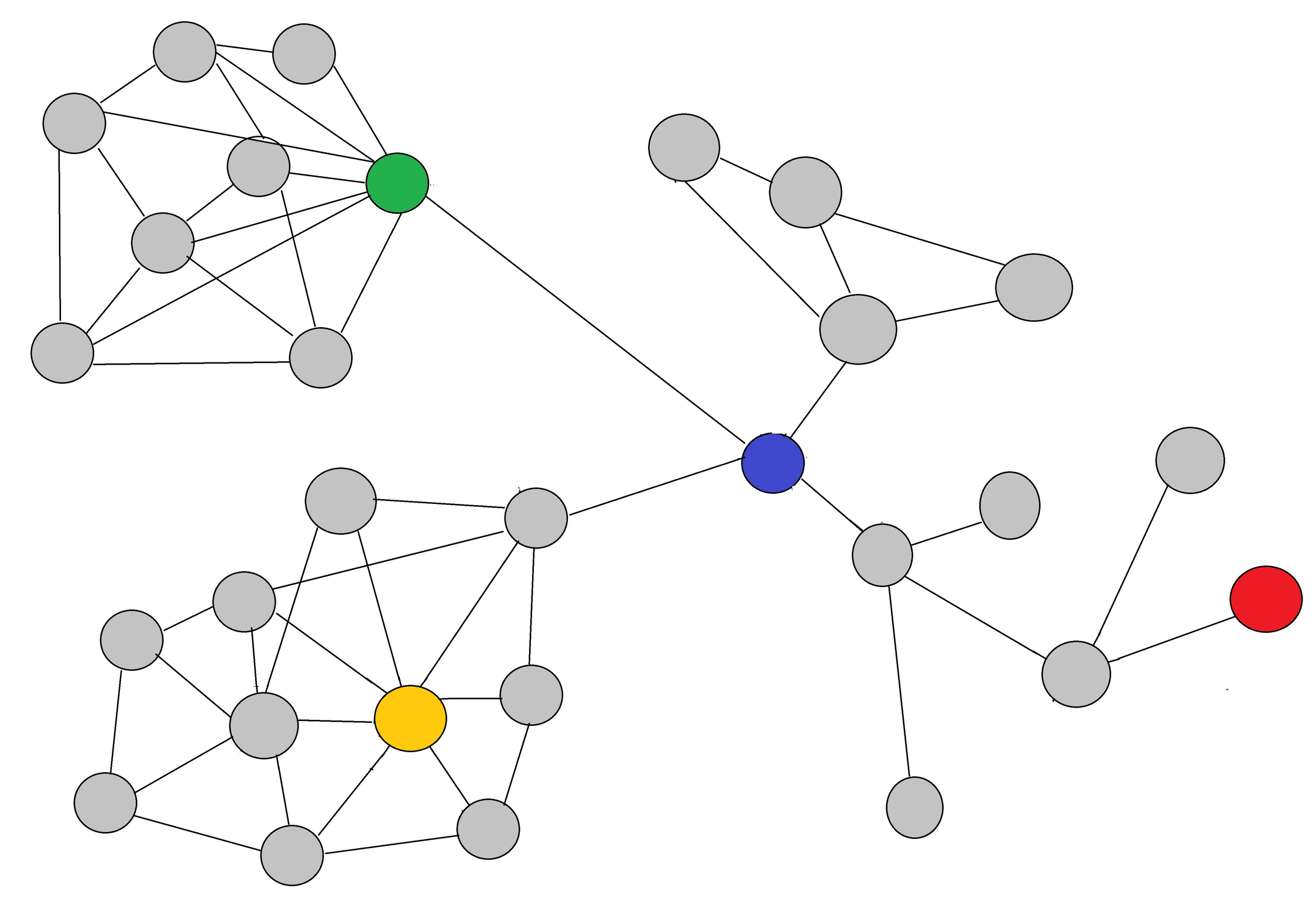

Supplement: Figure S1 — Network schematic. The network is presented as an undirected graph with nodes as protein/DEG (circle) and edges (line) indicating interactions between two connecting nodes. Node importance is estimated as degree centrality (the number of connections the node has to other nodes) and betweenness centrality (the number of shortest paths going through the node). Green represents nodes with both high degree and betweenness values. Blue represents nodes with a low degree but high betweenness value. Yellow represents nodes with a high degree but low betweenness value. Red represents nodes with a low degree and betweenness value. [file Image_1.TIF]

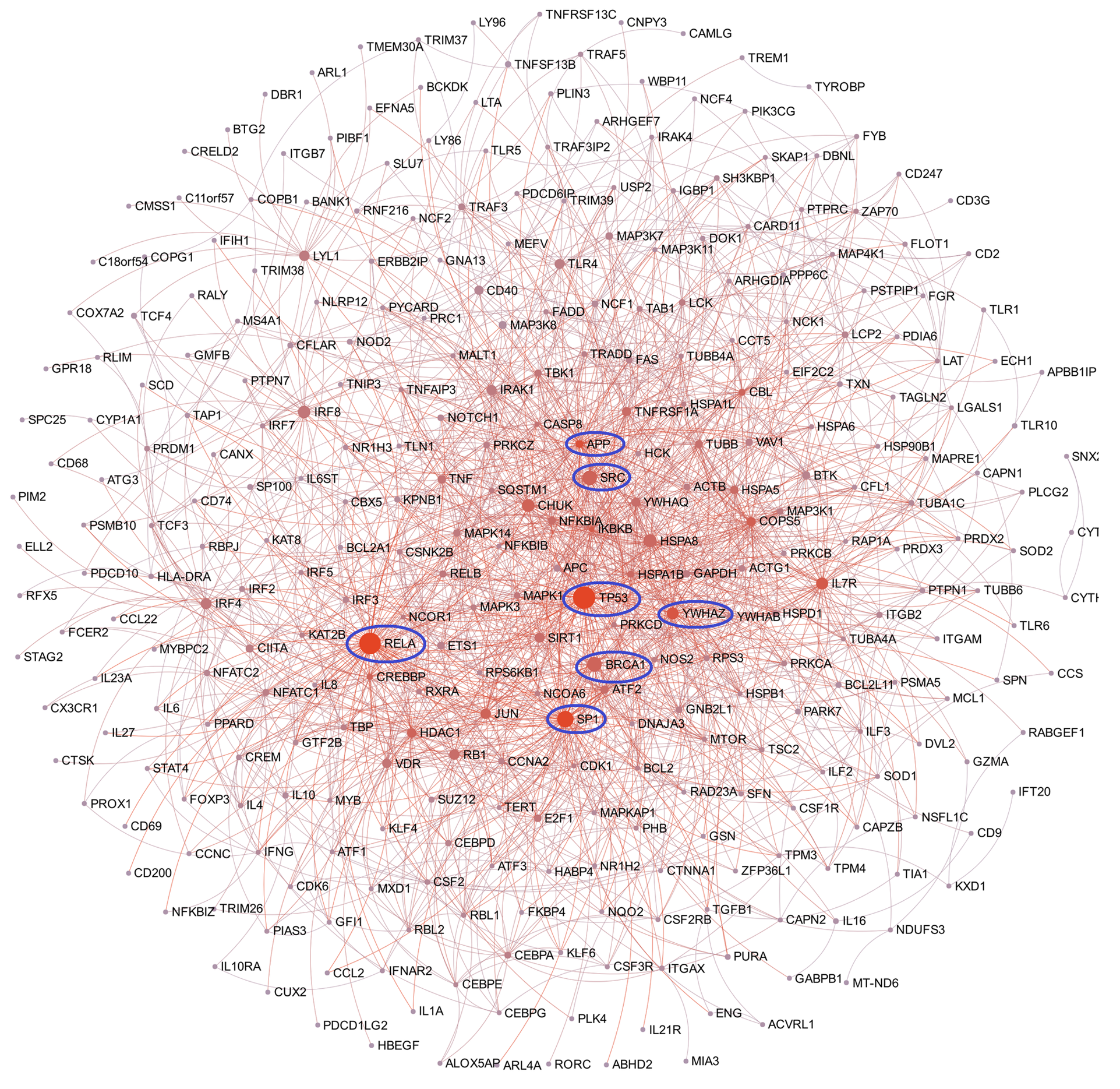

Supplement: Figure S2 — Topology of the peripheral blood mononuclear cell- associated Interactome (336 DEG, acute stage of infection). The network is presented as an undirected graph with nodes as protein/DEG (green arrows) and edges (blue arrows) indicating interactions between two connecting proteins. Node importance was estimated as degree centrality (the number of connections the node has to other nodes) and betweenness centrality (the number of shortest paths going through the node). Nodes with higher degree and betweenness values are darker red and larger, respectively. The top five nodes with the highest degree or betweenness values are highlighted with blue circles. [file Image_2.TIF]

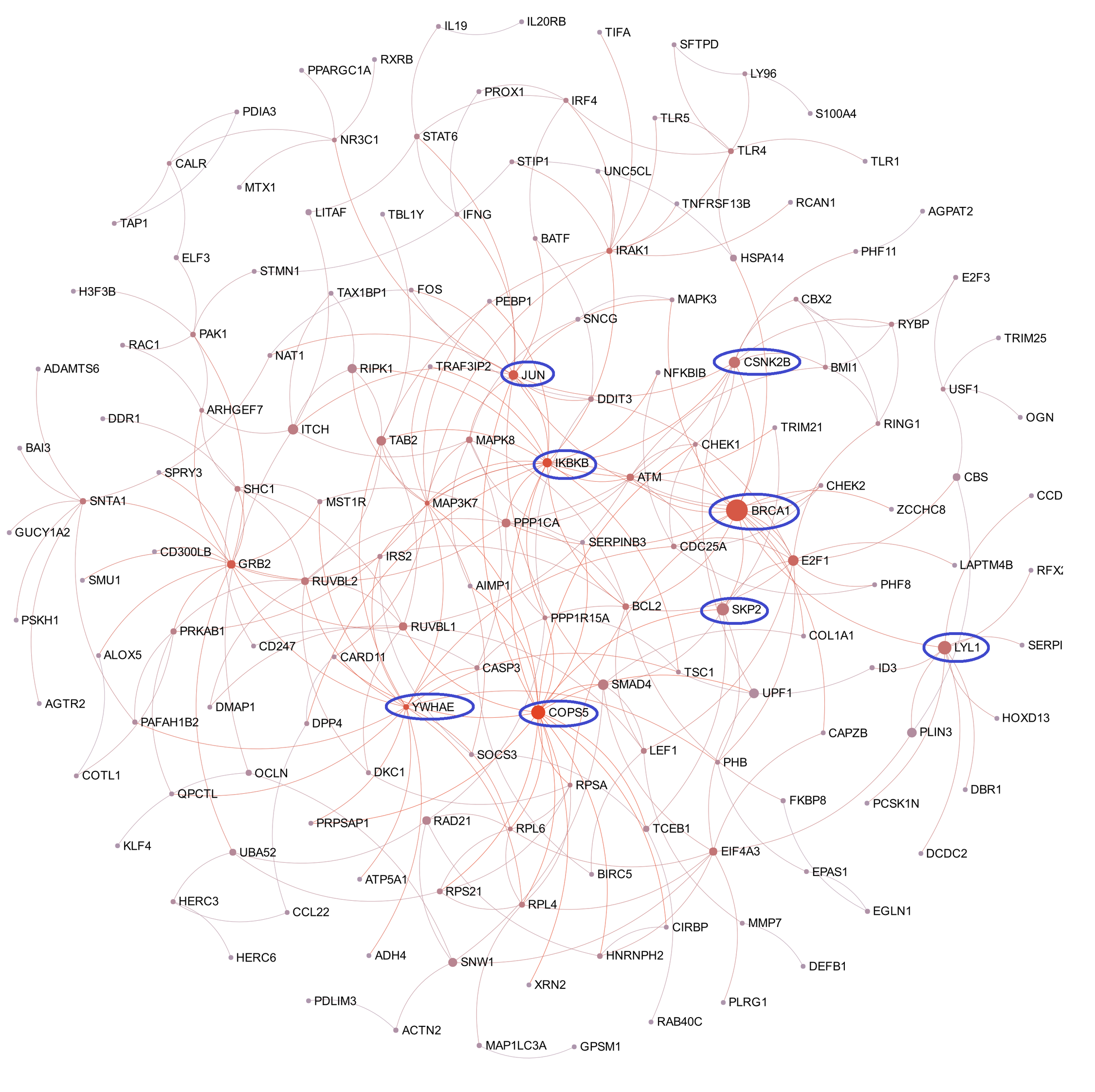

Supplement: Figure S3 — A topologic view of the network (186 DEG, Chronic stage of infection). Red and green nodes represent genes showing increased and decreased expression, respectively. The top five hub nodes with highest degree or betweenness values are highlighted with blue circles. [file Image_3.TIF]

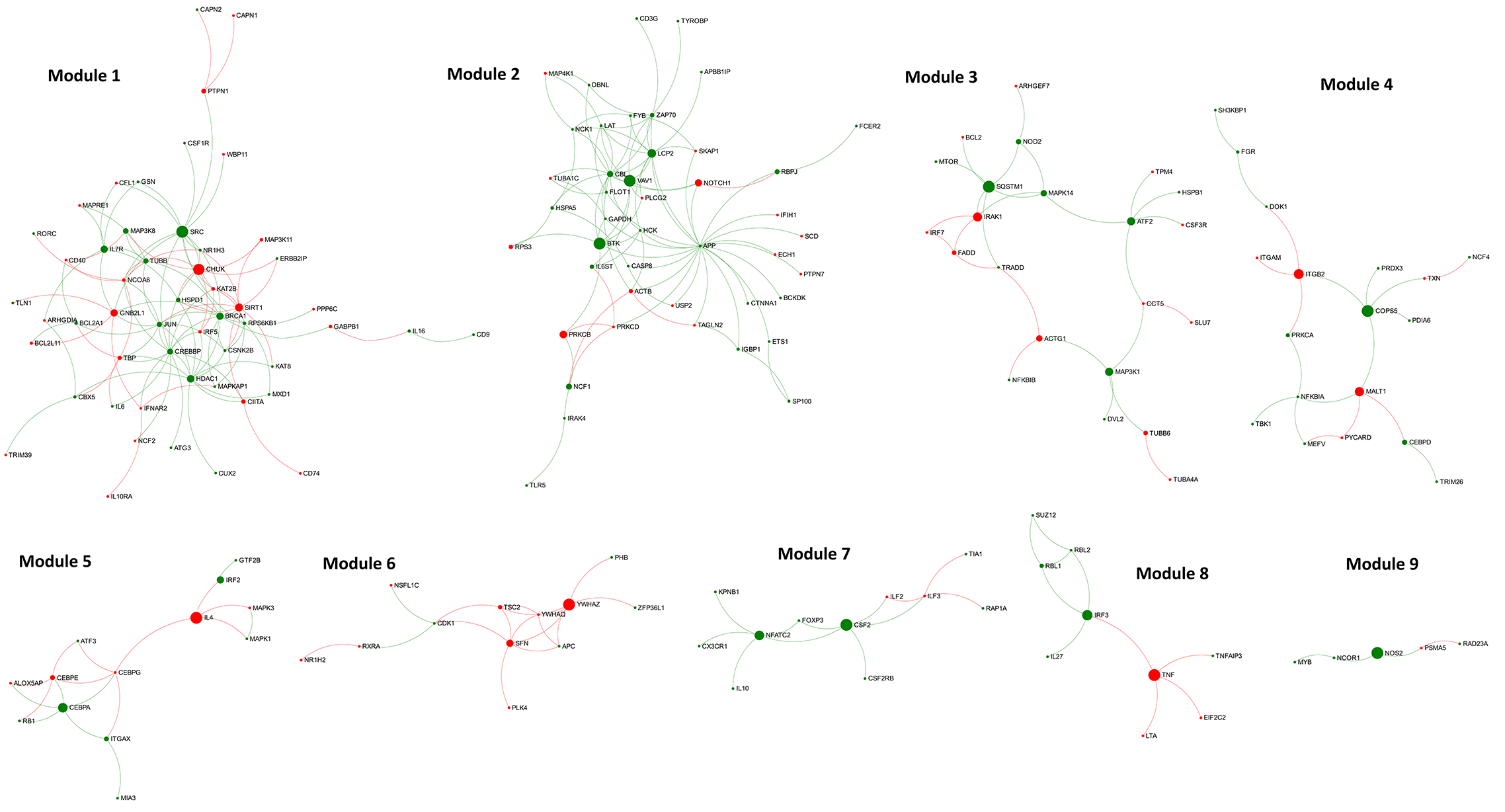

Supplement: Figure S4 — Expression pattern of nine modules extracted from the network representing acute stage of Fasciola hepatica infection. Red and green nodes represent genes showing increased/decreased expression, respectively. The size of nodes is proportional to their betweenness centrality values. The sequence number is ranked by module size. [file Image_4.TIF]

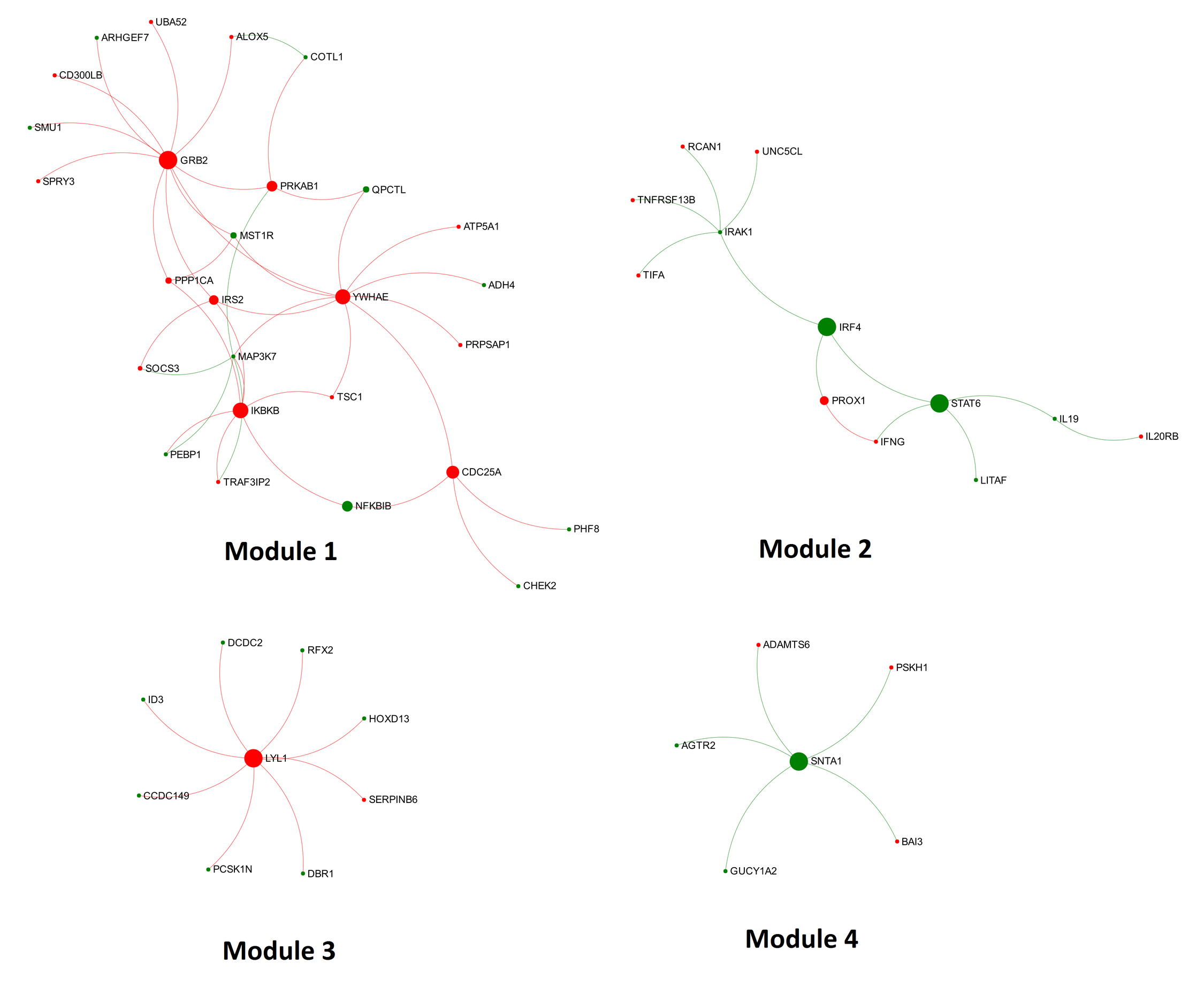

Supplement: Figure S5 — Expression patterns of four modules extracted from the network representing chronic stage of Fasciola hepatica infection. Red nodes are upregulated and green nodes are downregulated. The size of nodes is proportional to their betweenness centrality values. [file Image_5.TIF]
